# A Genetic Polymorphism in the *Pannexin1* Gene Predisposes for The Development of Endothelial Dysfunction with Increasing BMI

**DOI:** 10.3390/biom10020208

**Published:** 2020-01-31

**Authors:** Filippo Molica, Alessandra Quercioli, Fabrizio Montecucco, Thomas H. Schindler, Brenda R. Kwak, Sandrine Morel

**Affiliations:** 1 Department of Pathology and Immunology, University of Geneva,1211 Geneva, Switzerland; brenda.kwakchanson@unige.ch (B.R.K.); sandrine.morel@unige.ch (S.M.); 2 Department of Medical Specialities-Cardiology, University of Geneva, 1211 Geneva, Switzerland; alessandra.quercioli@libero.it (A.Q.); fabrizio.montecucco@unige.it (F.M.); thschindler@wustl.edu (T.H.S.)

**Keywords:** Panx1, polymorphism, endothelial function, obesity

## Abstract

Endothelial dysfunction worsens when body mass index (BMI) increases. Pannexin1 (Panx1) ATP release channels regulate endothelial function and lipid homeostasis in mice. We investigated whether the Panx1-400A>C single nucleotide polymorphism (SNP), encoding for a gain-of-function channel, associates with endothelial dysfunction in non-obese and obese individuals. Myocardial blood flow (MBF) was measured by ^13^N-ammonia positron emission/computed tomography at rest, during cold pressor test (CPT) or dipyridamole-induced hyperemia. Myocardial flow reserve (MFR) and endothelial function were compared in 43 non-obese (BMI < 30 kg/m^2^) vs. 29 obese (BMI ≥ 30 kg/m^2^) participants and genotyping for the Panx1-400A>C SNP was performed. Groups comprised subjects homozygous for the C allele (*n* = 40) vs. subjects with at least one A allele (*n* = 32). MBF (during CPT or hyperemia), MFR and endothelial function correlated negatively with BMI in the full cohort. BMI correlated negatively with MFR and endothelial function in non-obese Panx1-400C subjects, but not in Panx1-400A individuals nor in obese groups. BMI correlated positively with serum triglycerides, insulin or HOMA. MFR correlated negatively with these factors in non-obese Panx1-400C but not in Panx1-400A individuals. Here, we demonstrated that Panx1-400C SNP predisposes to BMI-dependent endothelial dysfunction in non-obese subjects. This effect may be masked by excessive dysregulation of metabolic factors in obese individuals.

## 1. Introduction

Endothelial dysfunction, the main cause of coronary artery disease, aggravates with increasing bodyweight and obesity [1]. A possible role for ATP in the process of endothelial dysfunction is still a matter of debate. Extracellular nucleotides, such as ATP, generally display vasculoprotective properties by regulating vasodilation through nitric oxide production [2,3]. However, ATP has also been shown to participate to the onset of endothelial dysfunction through the activation of P2X4 and P2X7 purinergic receptors [4]. In fact, prolonged exposure of endothelial cells to ATP enhances the expression of chemokines and adhesion molecules by endothelial cells [4,5]. Pannexin1 (Panx1) channels are newly discovered ATP release channels that are increasingly recognized to critically regulate vascular function [6]. Panx1 is expressed in most cells of the body, including diverse types of blood cells, endothelial and smooth muscle cells but also adipocytes [7,8]. ATP released through Panx1 channels can target surface receptors in a paracrine or autocrine fashion allowing for intercellular signaling and tissue homeostasis. Panx1 channels are activated under specific conditions such as mechanical stretch, elevated extracellular potassium or metabotropic (P2Y) or ionotropic (P2X) receptor activation, resulting in the elevation of intracellular calcium. Studies performed in mice have revealed a key role for Panx1 channels in the regulation of endothelial function as well as for lipid homeostasis. Thus, Panx1 has been shown to facilitate endothelium-dependent vasodilation in large arteries [9]. During acute inflammation, Panx1 channels in the endothelium promote leukocyte adhesion and emigration through the vascular wall [10,11]. Moreover, Panx1 deficiency has been shown to be associated with atherosclerosis and white adipose tissue accumulation as well as with lymphatic dysfunction [12]. In mice, Panx1 appeared to control adipose stromal cell proliferation and differentiation, thereby regulating fat accumulation in vivo [13]. Interestingly, a Panx1-400A>C single nucleotide polymorphism (SNP; rs1138800), inducing an amino acid change from a Glutamine to a Histidine (Q5H) in the N-terminus of Panx1, encodes for a gain-of-function channel [14]. The Panx1-400C genetic variant, encoding for the gain-of-function channel, specifically enhanced collagen-induced ATP release and platelet aggregation [14]. Although multiple studies in mice point to a role for Panx1 in vascular pathologies caused by impaired endothelial function, whether the Panx1-400A>C SNP associates with endothelial dysfunction in humans is presently unknown.

## 2. Materials and Methods

### 2.1. Study Population

Study participants have been recruited at the Geneva University Hospitals by flyers and newspaper advertisements. The study was approved by the institutional review board of the Geneva University Hospitals (No. 07-183) [15,16]. Each participant signed an approved informed consent form. Study participants underwent an initial screening visit comprising physical examination, electrocardiogram, blood pressure measurements and routine blood chemistry in a fasting state. Then, they underwent ^13^N-ammonia positron emission tomography (PET)/computed tomography (CT) [17] measurements of myocardial blood flow (MBF) at rest and during vasomotor stress performed in a fasting state to assess coronary circulatory function.

Physical examination revealed normal findings in all applicants and resting electrocardiograms showed also no abnormalities. Inclusion criteria were absence of arterial hypertension (blood pressure ≤ 140/90 mmHg) or diabetes mellitus (fasting plasma glucose obtained on more than two occasions ≤ 126 mg/dL), a non-smoker status and a normal stress-rest perfusion imaging on ^13^N-ammonia PET/CT, which widely excluded the presence of flow limiting coronary artery disease lesions. The exclusion criteria were any cardiac or vasoactive medication, a history of variant angina, a family history of premature coronary artery disease, or clinically manifested cardiovascular or systemic diseases.

Recruited volunteers had a body mass index (BMI, kg/m^2^) between 20.4 and 51.1. According to subject’s BMI, two groups of participants were defined as 1) Non-obese group: 43 non-obese individuals with BMI < 30 kg/m^2^, and 2) Obese group: 29 individuals with BMI ≥ 30 kg/m^2^.

### 2.2. Blood Measurements

Blood chemistry included plasma triglycerides, total cholesterol, high-density lipoproteins (HDL), low-density lipoproteins (LDL), glucose, hemoglobin A1c (HbA1c), insulin and high-sensitive C-reactive protein (CRP) concentrations, as previously reported [15,16]. Assessment of insulin resistance using the homeostasis model assessment (HOMA) was calculated as fasting insulin (mIU/L) × fasting glucose (mg/dL)/22.5, as previously described [17].

### 2.3. Assessment of Myocardial Perfusion with PET/CT

Myocardial perfusion was determined by ^13^N-ammonia PET/CT as previously described [17]. Myocardial blood flow (MBF, mL/g/min) was assessed at rest, during a cold pressor test (CPT), and during hyperemia pharmacologically induced with standard infusion of dipyridamole (140 mg/kg/min). The endothelial function was defined as ΔMBF to CPT from rest, and the myocardial flow reserve (MFR) was defined as the ratio between MBF during hyperemia and MBF at rest, as previously described [16].

### 2.4. Phenotype–Genotype Association Studies

Polymerase chain reaction (PCR) for the Panx1-400A>C SNP was performed on DNA isolated from the blood of each study participant with the following primers (5′-to-3′): forward CCGGTGACTGGGTGAAGG, reverse GTCCTGGGCGAGGCTTAC, as previously described [14]. Sequencing of PCR products was performed at Fasteris (Geneva, Switzerland). To identify the *Panx1* genotype, all sequences comprising parts of the *Panx1* coding region were analyzed using the multiple sequence alignment program ClustalW and allele frequency was calculated (i.e., minor allele frequency = (WM + 2 × MM)/((WW + WM + MM) × 2)). Minor allele frequency was in accordance to the one obtained in an earlier study investigating Panx1-400A>C SNP [14] and the distribution of participants of each genotype was in the Hardy–Weinberg equilibrium. Similar to earlier studies [14,18], groups were divided in subjects homozygous for the C allele (Panx1-400C, *n* = 40) vs. subjects with at least one A allele (Panx1-400A, *n* = 32).

### 2.5. Statistical Analysis

In tables, results are shown as mean ± standard error of the mean (SEM) or as median (interquartile range). Comparisons of means were performed using ANOVA test and post-hoc Sidak’s multiple comparison test. Comparisons of medians were performed using non-parametric Kruskal–Wallis test and post-hoc Dunn’s multiple comparison test. Comparisons of distributions were performed using *χ*^2^ test. In figures, results are shown as individual values and as correlation. Spearman correlations were performed to examine association between variables. Differences were considered statistically significant at values of *p* < 0.05. Statistical analyses were performed using Graphpad Prism 8 software (GraphPad Software, Inc., San Diego, CA, USA).

## 3. Results

### 3.1. BMI Correlates with Cardiovascular Risk Factors

**Figure 1:**
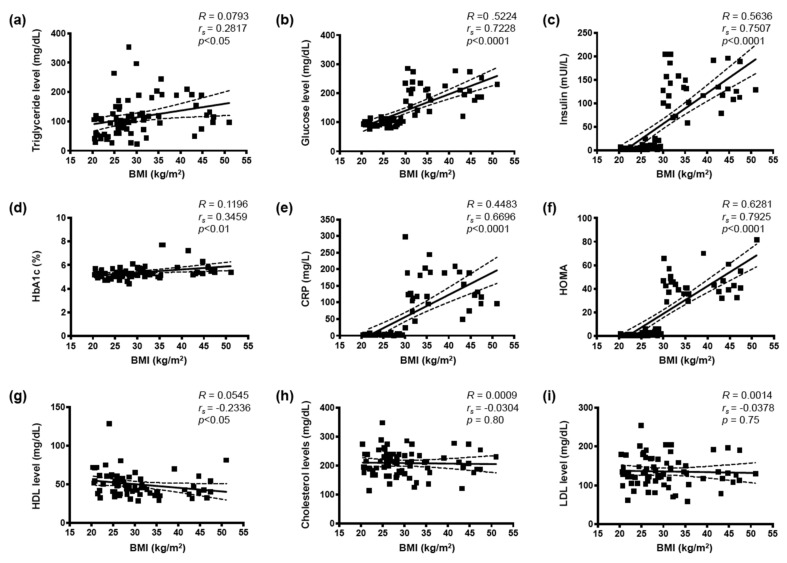
Body mass index (BMI) associates with cardiovascular risk factors. Spearman correlations between BMI and serum triglycerides (**a**), glucose (**b**), insulin (**c**), hemoglobin A1c (HbA1**c**, (**d**)), C-reactive protein (CRP, (**e**)), homeostasis model assessment (HOMA) (**f**), high density-lipoproteins (HDL, g), total cholesterol (**h**), and low-density lipoproteins (LDL) (**i**), in the full cohort of participants (*n* = 72).

The clinical characteristics and laboratory measurements among the groups studied are shown in Table 1. Spearman correlations showed that in the full cohort BMI was positively correlated with cardiovascular risk factors known to be predictors of CPT-induced ΔMBF and hyperemic MBF during dipyridamole stimulation [15] such as serum triglycerides (Figure 1a), glucose (Figure 1b), insulin (Figure 1c), HbA1c (Figure 1d) and CRP (Figure 1e). BMI was also positively correlated with HOMA as shown in Figure 1f. As expected, BMI was negatively correlated with the serum HDL concentration (Figure 1g). There was no correlation between BMI and total cholesterol (Figure 1h) or LDL (Figure 1i) serum levels. These results show that, in our cohort of 72 participants, increased BMI correlates with cardiovascular risk factors, which is in accordance with previously published studies [19,20,21].

### 3.2. BMI, Hemodynamic Parameters, and Panx1-400A>C SNP

**Figure 2:**
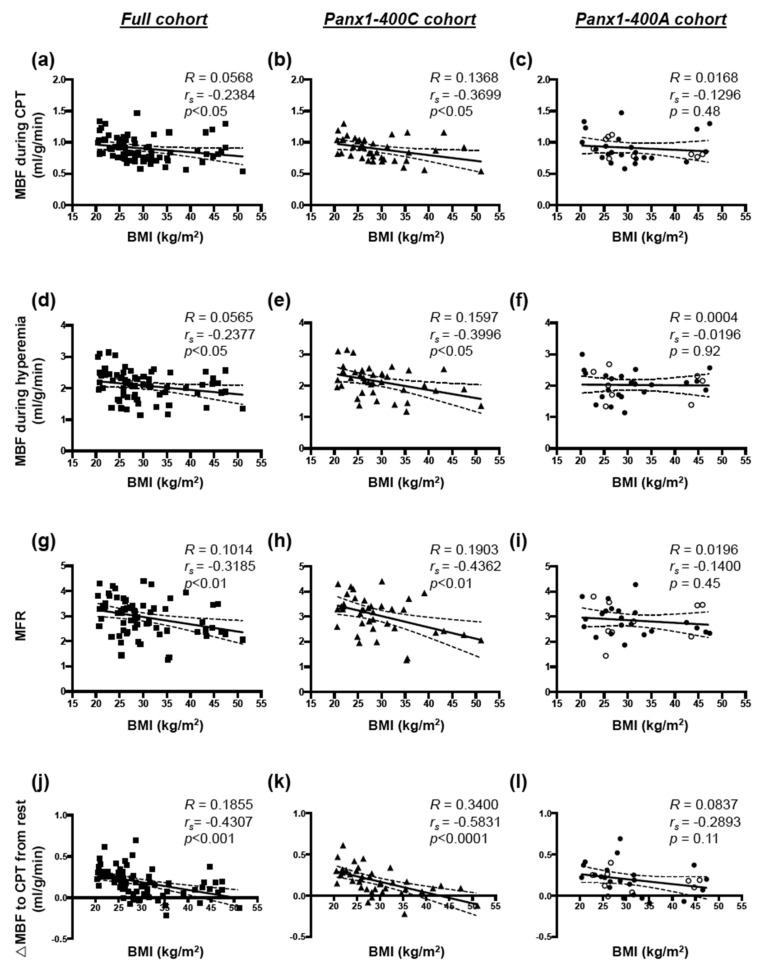
BMI, hemodynamic parameters and Panx1-400A>C SNP. Spearman correlations between body mass index (BMI) and myocardial blood flow (MBF) during cold pressor test (CTP) (**a**–**c**), MBF during hyperemia (**d**–**f**), myocardial flow reserve (MFR) (**g**–**i**) and endothelial function (ΔMBF to CPT from rest, **j**–**l**) in the full cohort ((**a**,**d**,**g,j**) *n* = 72), the Panx1-400C cohort (**b**,**e**,**h,k**) *n* = 40) and the Panx1-400A cohort (**c**,**f**,**i,l**) *n* = 32). AA genotype: open circles; AC genotype: closed circles.

When analyzed separately, the three cohorts did not show differences in the age and the proportion of females/males (Table 1). In addition, no differences were found for the height, the weight and the BMI (Table 1). Moreover, heart rate, systolic and diastolic blood pressure values at rest were not different in the three cohorts (Table 1). Whereas MBF at rest was not affected by BMI (data not shown), MBF during CPT, MBF during hyperemia, MFR, and endothelial function were negatively correlated with BMI in the full cohort (Figure 2a,d,g,j, respectively). Mean values of all these parameters were not different between the full, the Panx1-400C and the Panx1-400A cohorts (Table 2). In the Panx1-400C cohort, MBF during CTP (Figure 2b), MBF during hyperemia (Figure 2e), MFR (Figure 2h) and endothelial function (Figure 2k) were also negatively correlated with BMI whereas no correlation between these hemodynamic parameters and BMI was found in the Panx1-400A cohort (Figure 2c,f,i,l).

MBF at rest, MBF during hyperemia and MFR were not different between non-obese (BMI < 30 kg/m^2^) and obese (BMI ≥ 30 kg/m^2^) subjects in the three cohorts and were not affected by the Panx1-400A>C SNP (Table 3). During CPT, MBF was reduced in obese subjects in comparison to non-obese individuals in the full cohort (Table 3). A similar trend was observed in the Panx1-400C cohort but not in the Panx1-400A cohort (Table 3). Mean endothelial function was reduced in obese individuals in comparison to non-obese subjects in all three cohorts (Table 3).

**Figure 3:**
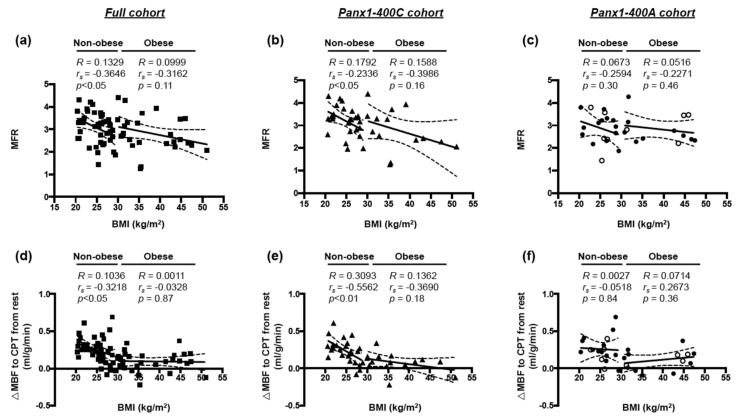
Correlation between BMI and MFR or endothelial function. Spearman correlations between body mass index (BMI) and myocardial flow reserve (MFR, (**a**–**c**)) or endothelial function (∆MBF to CPT from rest, (**d–f**)) in non-obese and obese subjects in the full cohort ((**a**,**d**) *n* = 43 and 29, respectively), the Panx1-400C cohort ((**b**,**e**) *n* = 25 and 15, respectively) and the Panx1-400A cohort ((**c**,**f**) *n* = 18 and 14, respectively). AA genotype: open circles; AC genotype: closed circles.

In non-obese individuals, negative correlations between BMI and MFR and between BMI and endothelial function were observed in the full cohort of participants (Figure 3a,d, respectively). Interestingly, such correlations for non-obese subjects were also observed in the Panx1-400C cohort (Figure 3b,e) but not in the Panx1-400A cohort (Figure 3c,f). No correlation between BMI and MFR or between BMI and endothelial function was observed for obese subjects in the three cohorts (Figure 3).

Altogether, these results bring out that the Panx1-400A>C SNP does not change by itself hemodynamic parameters but that the impairment of these hemodynamic parameters is correlated with increased BMI in Panx1-400C subjects but not in Panx1-400A individuals. Moreover, this effect of the Panx1-400C SNP on the impairment of these hemodynamic parameters with increasing bodyweight is only present in non-obese subjects.

### 3.3. Correlation between BMI and Cardiovascular Risk Factors in Non-obese and Obese Subjects

**Figure 4:**
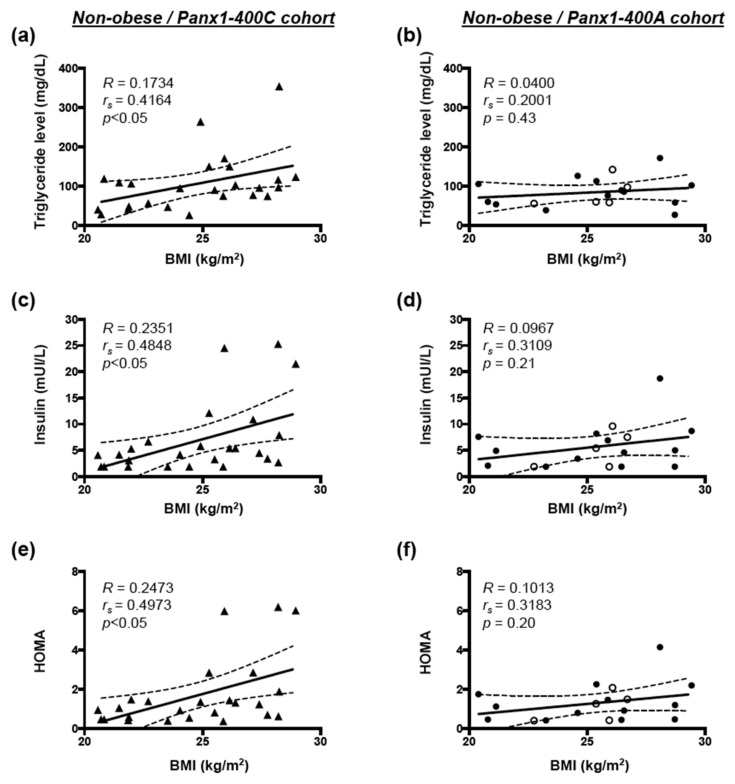
Correlation between BMI and cardiovascular risk factors in non-obese subjects. Spearman correlations in non-obese subjects between body mass index (BMI) and serum triglycerides (**a**,**b**), insulin (**c**,**d**) and HOMA (**e**,**f**) in the Panx1-400C cohort ((**a**,**c**,**e**) *n* = 25) and the Panx1-400A cohort ((**b**,**d**,**f**) *n* = 18). AA genotype: open circles; AC genotype: closed circles.

In non-obese individuals, serum triglycerides and insulin as well as HOMA were positively correlated with increasing BMI in the Panx1-400C cohort (Figure 4a,c,e respectively), but not in the Panx1-400A cohort (Figure 4b,d,f, respectively). In both cohorts, no correlation was observed between increasing BMI and serum glucose, HbA1c, CRP, or HDL concentrations for non-obese subjects (data not shown). For obese subjects, no correlation was shown between increasing BMI and the various cardiovascular risk factors tested (data not shown).

Here, we show that in non-obese subjects, Panx1-400C SNP favors positive correlation between BMI and triglyceride levels, insulin levels and HOMA, which is not the case for non-obese Panx1-400A individuals.

### 3.4. Correlation Between Hemodynamic Parameters and Cardiovascular Risk Factors in Non-Obese Subjects

**Figure 5:**
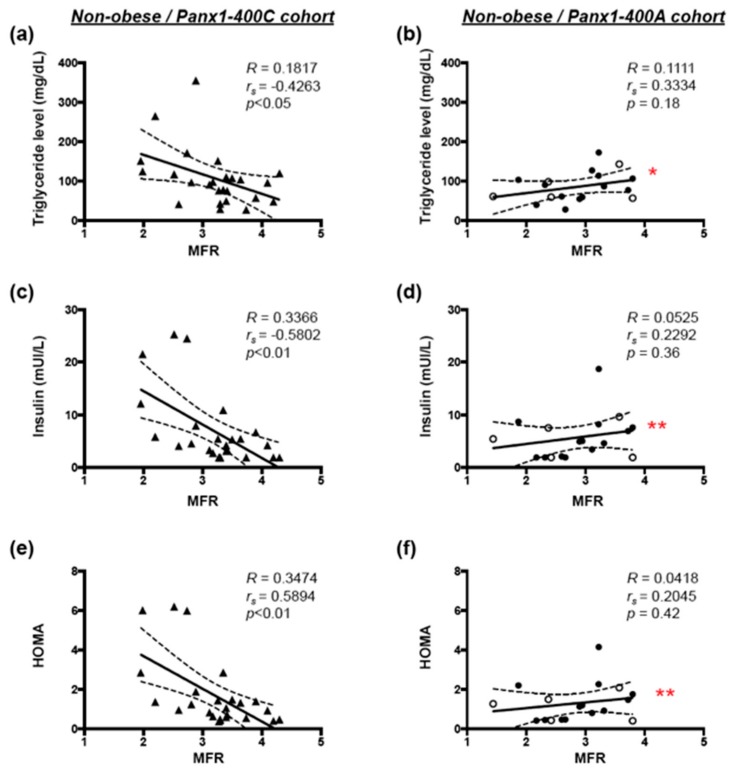
Correlation between MFR and cardiovascular risk factors in **n**on-obese subjects. Spearman correlations in non-obese individuals between myocardial flow reserve (MFR) and serum triglycerides (**a**,**b**), insulin (**c**,**d**), and HOMA (**e**,**f**) in the Panx1-400C cohort ((**a**,**c**,**e**) *n* = 25) and the Panx1-400A cohort ((**b**,**d**,**f**) *n* = 18). AA genotype: open circles; AC genotype: closed circles. * *p* < 0.05; ** *p* < 0.01: slope of non-obese subjects in the Panx1-400C cohort vs. slope of non-obese subjects in the Panx1-400A cohort.

Negative correlations between MFR and serum triglycerides, insulin or HOMA were observed in non-obese subjects from the Panx1-400C cohort (Figure 5a,c,e, respectively). These correlations were completely absent in non-obese subjects from the Panx1-400A cohort (Figure 5b,d,f). Moreover, the slopes of the correlation between MFR and these cardiovascular risk factors in non-obese individuals were significantly different between the Panx1-400C and the Panx1-400A cohorts (Figure 5a vs. Figure 5b, Figure 5c vs. Figure 5d, and Figure 5e vs. Figure 5f).

**Figure 6:**
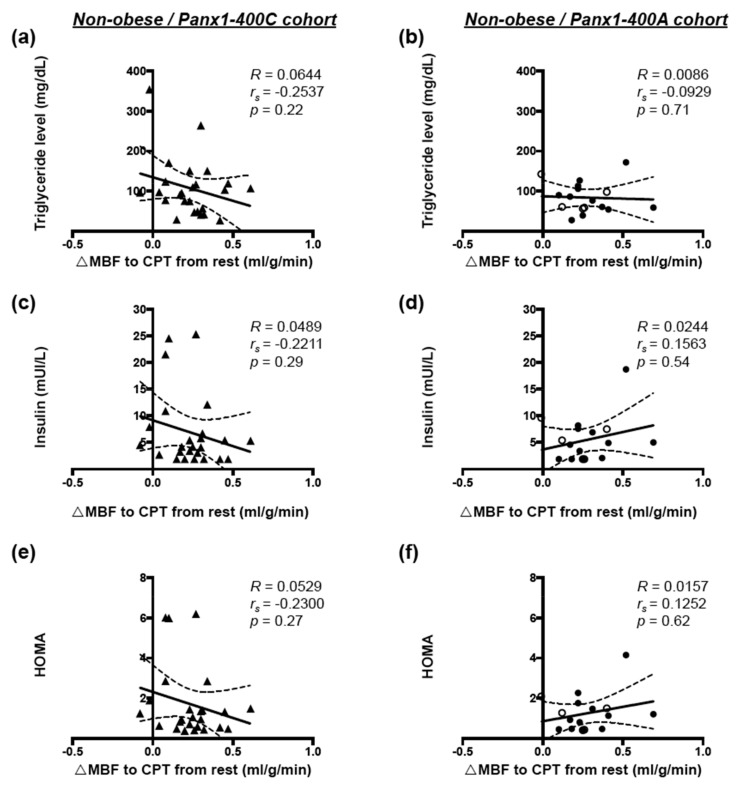
Correlation between endothelial function and cardiovascular risk factors in non-obese subjects. Spearman correlations in non-obese subjects between endothelial function and serum triglycerides (**a,b**), insulin (**c**,**d**), and HOMA (**e**,**f**) in the Panx1-400C cohort ((**a**,**c**,**e**) *n* = 25) and the Panx1-400A cohort ((**b**,**d**,**f**) *n* = 18). AA genotype: open circles; AC genotype: closed circles.

Finally, no correlation was found between endothelial function and serum triglycerides (Figure 6a,b), insulin (Figure 6c,d) or HOMA (Figure 6e,f) in non-obese subjects from the Panx1-400C cohort (Figure 6a,c,e) or the Panx1-400A cohort (Figure 6b,d,f).

Altogether, our results demonstrate that for non-obese Panx1-400C subjects, elevation in triglyceride and insulin concentrations as well as increased HOMA promote endothelial dysfunction and a reduction in MFR, effects that are absent or even slightly inverted in non-obese Panx1-400A subjects.

## 4. Discussion

The present study shows for the first time an implication of the Panx1-A400C SNP in endothelial dysfunction and reduced myocardial perfusion under stress. Panx1 channels are involved in physiological and pathological processes ascribed to ATP release such as the regulation of vasomotor function, glucose metabolism, leukocyte recruitment, adipogenic progenitor cell differentiation, ischemia–reperfusion injury or pain [22,23,24,25,26,27,28,29]. This multiplicity of functions involves different mechanisms of Panx1 channel activation including elevated extracellular potassium, stretch, Src family kinase-mediated phosphorylation, or cleavage of the C-terminal tail by caspases [8]. The SNP studied in the present study has been demonstrated to affect Panx1 channel functionality [14]. The base pair modification A400C in the *Panx1* gene results in an amino acid substitution at position 5 in the cytoplasmic N-terminal part of the protein. This shift from an uncharged Glutamine to a positively charged Histidine may induce conformational changes in the pore formed by the hexameric assembly of Panx1 proteins, leading to enhanced ATP release. Indeed, *Panx1*-deficient CHO cells transfected with Panx1-400C displayed increased basal and stimulated ATP release compared to cells transfected with Panx1-400A [14]. The effects of other N-terminal modifications on Panx1 channel opening have been demonstrated by patch clamp experiments showing that a weak voltage-dependent activity is drastically increased when a Glycine–Serine amino acid pair is inserted after the first Methionine [30].

The Panx1-A400C SNP has previously been described as potential modifier of atherothrombosis [14]. Indeed, the gain-of-function channel variant (Panx1-400C) was associated with enhanced collagen-induced platelet reactivity in a cohort of 96 healthy volunteers and was also identified in cohorts of patients with a hyper- or hypo-reactive platelet phenotype with the Panx1-400C variant being more prevalent in the hyper-reactive patients. Here, we aimed to evaluate the potential implication of this *Panx1* genetic variant in endothelial dysfunction and myocardial perfusion in obese and non-obese individuals. Overweight and obesity are conditions known to alter endothelial cell functionality [1] through mechanisms such as insulin resistance, increased serum cholesterol and LDL levels, pro-inflammatory adipokine release and elevated free fatty acids (FFAs) production by adipose tissue [31]. In accordance, we showed in our full cohort of 72 study participants that increased BMI was positively correlated with serum triglycerides, glucose, insulin, HbA1c and CRP levels, and with insulin resistance (HOMA) (Figure 1). Moreover, increased BMI was negatively correlated with myocardial perfusion under stress and with endothelial function (Figure 2). These negative correlations were also found in a subgroup of non-obese individuals but were lost in obese subjects (Figure 3). Interestingly, similar negative correlations were observed in Panx1-400C subjects but not in Panx1-400A individuals (Figure 2 and Figure 3). In Panx1-400C non-obese subjects, BMI was positively correlated with triglycerides and insulin levels and with HOMA (Figure 4). Such positive correlations were absent in Panx1-400A non-obese individuals. Elevated triglyceride levels as well as increased glucose and insulin concentrations are known to induce endothelial dysfunction by increasing inflammation, apoptosis, and mainly by increasing reactive oxygen species production [32,33,34]. The latter processes have all three been shown to be affected by Panx1 expression or Panx1 channel functionality. For instance, in atherosclerosis-prone mice fed a high cholesterol diet, ubiquitous *Panx1* deletion was shown to reduce serum triglyceride and FFA levels [12]. This effect was attributed to impairment of lymphatic function in *Panx1*-deficient mice, which is affecting lipid uptake as demonstrated with an oral lipid tolerance test. Moreover, experiments performed in rat INS-1E beta-cells showed that Panx1 channel activation by chronic fructose exposure potentiated glucose-stimulated insulin secretion [29]. In addition, Panx1 channel activity appeared to contribute to the control of metabolic homeostasis by regulating insulin-stimulated glucose uptake in adipocytes [24]. Finally, mice with ubiquitous *Panx1* deletion have increased fat mass and reduced lean mass [12,13]. This increase in body adiposity is mainly due to larger volumes of subcutaneous and visceral adipose tissues [12] and results in increased glucose and insulin levels [13]. Altogether, these observations suggest strong links between Panx1 channel functionality, metabolism and endothelial function. Our data further strengthen this hypothesis as we found that myocardial perfusion under stress was negatively correlated with BMI (Figure 3), serum triglycerides, insulin levels and HOMA (Figure 5) in Panx1-400C non-obese subjects expressing the gain-of-function channel, while no correlation between these parameters was found in the non-obese Panx1-400A cohort. The reason that the effect of the gain-of-function channel is lost in obese subjects might be an exacerbated increase in metabolic and cardiovascular risk factors in this sub-population. Altogether, these results suggest that the Panx1-A400C SNP by itself does not influence hemodynamic parameters, but that the impairment of hemodynamic function is correlated with increased BMI in Panx1-400C subjects but not in Panx1-400A individuals. Many studies indicate that the risk to develop endothelial dysfunction increases with the number of risk factors co-existing in an individual [35,36]. This association between increased BMI and reduced MFR in the non-obese subjects of the Panx1-400C cohort could be attributed to increased risk factors for endothelial dysfunction. Although no significant correlation between endothelial function and serum triglycerides, insulin and HOMA were observed in Panx1-400C individuals, it is noteworthy that a similar trend to the one obtained for myocardial perfusion under stress was found (Figure 6). Although the main limitation of the present study is the small size of the cohorts, to the best of our knowledge, this study is the first one uniquely linking the Panx1 SNP, encoding for a gain-of-function ATP release channel, BMI, cardiovascular risk factors, endothelial function and myocardial perfusion. The prevalent Panx1-400C SNP thus drives the relation between impaired hemodynamic parameters, increased occurrence of cardiovascular risk factors and increased BMI in the general population, which may be taken into account in future investigations.

## 5. Conclusions

Our data show that the Panx1-400C SNP predisposes for the development of endothelial dysfunction and reduced myocardial perfusion during stress (induced by CPT or dipyridamole-induced hyperemia) with increasing BMI in non-obese subjects.

## Figures and Tables

**Table 1:** Patient characteristics at the time of the inclusion in the study.

	Full Cohort (*n* = 72)	Panx1-400C Cohort (*n* = 40)	Panx1-400A Cohort (*n* = 32)	*p-*Value
Age, years, mean ± SEM	43 ± 1	45 ± 2	42 ± 2	0.63
Female/male, *n* (%)	19 (26)/53 (74)	10 (25)/30 (75)	9 (28)/23 (72)	0.89
Weight (kg), mean ± SEM	90 ± 3	88 ± 3	93 ± 5	0.58
Height (cm), mean ± SEM	173 ± 1	173 ± 1	173 ± 2	0.98
BMI (kg/m^2^), mean ± SEM	30 ± 1	45 ± 2	31 ± 1	0.58
Heart rate at rest (b.p.m.),median (IQR)	63 (56–70)	63 (55–70)	64 (58–70)	0.97
SBP at rest (b.p.m.),median (IQR)	119 (110–129)	118 (106–126)	120 (112–132)	0.28
DBP at rest (b.p.m.),median (IQR)	74 (68–80)	73 (67–80)	76 (68–83)	0.75

BMI: body mass index; b.p.m: beat per minute; DBP: diastolic blood pressure; IQR: interquartile range; SBP: systolic blood pressure; SEM: standard error of the mean.

**Table 2:** MBF, MFR, and endothelial function measured during ^13^N-ammonia PET/CT in the three cohorts.

	Full Cohort (*n* = 72)	Panx1-400C Cohort (*n* = 40)	Panx1-400A Cohort (*n* = 32)	*p-*Value
MBF at rest (mL/g/min), median (IQR)	0.72 (0.62–0.78)	0.72 (0.63–0.76)	0.72 (0.62–0.79)	0.99
MBF during CPT (mL/g/min), median (IQR)	0.85 (0.76–1.04)	0.90 (0.75–1.04)	0.84 (0.76–1.05)	0.99
MBF during hyperemia (mL/g/min), median (IQR)	2.11 (1.79–2.42)	2.15 (1.84–2.44)	2.08 (1.71–2.32)	0.59
MFR, median (IQR)	3.03 (2.41–3.43)	3.28 (2.52–3.50)	2.76 (2.38–3.31)	0.40
ΔMBF to CPT from rest (mL/g/min), mean ± SEM	0.186 ± 0.020	0.179 ± 0.026	0.194 ± 0.030	0.93

CPT: cold pressor test; CT: computed tomography; IQR: interquartile range; MBF: myocardial blood flow; MFR: myocardial flow reserve; ΔMBF to CPT from rest: endothelial function; PET: positron emission tomography; SEM: standard error of the mean.

**Table 3:** MBF, MFR, and endothelial function measured during ^13^N-ammonia PET/CT in non-obese and obese subjects from the three cohorts.

	Full Cohort Non-Obese (*n* = 43)	Full Cohort Obese (*n* = 29)	*p*-Value	*Panx1-400C* Non-Obese (*n* = 25)	*Panx1-400C* Obese (*n* = 15)	*p*-Value	*Panx1-400A* Non-Obese (*n* = 18)	*Panx1-400A* Obese (*n* = 14)	*p*-Value
MBF at rest (mL/g/min), median (IQR)	0.72 (0.63–0.76)	0.74 (0.62–0.84)	0.35	0.72 (0.65–0.75)	0.69 (0.57–0.84)	0.74	0.70 (0.60–0.79)	0.75 (0.63–0.80)	0.42
MBF during CPT (mL/g/min), median (IQR)	0.93 (0.81–1.05)	0.78 (0.71–0.92)	<0.01	0.93 (0.83–1.04)	0.76 (0.70–0.93)	0.06	0.92 (0.78–1.10)	0.80 (0.75–0.87)	0.12
MBF during hyperemia (mL/g/min), median (IQR)	2.18 (1.71–2.44)	2.08 (1.84–2.31)	0.43	2.24 (1.98–2.47)	1.95 (1.44–2.41)	0.14	1.94 (1.59–2.40)	2.10 (1.95–2.23)	0.51
MFR, median (IQR)	3.22 (2.6–3.5)	2.69 (2.34–3.42)	0.16	3.3 (2.78–3.58)	2.64 (2.22–3.49)	0.20	2.92 (2.36–3.38)	2.69 (2.36–3.30)	0.70
ΔMBF to CPT from rest (mL/g/min), mean ± SEM	0.246 ± 0.025	0.095 ± 0.023	<0.001	0.238 ± 0.032	0.081 ± 0.033	<0.01	0.259 ± 0.042	0.111 ± 0.035	<0.05

CPT: cold pressor test; CT: computed tomography; IQR: interquartile range; MBF: myocardial blood flow; MFR: myocardial flow reserve; ΔMBF to CPT from rest: endothelial function; PET: positron emission tomography; SEM: standard error of the mean.
